# Supplementary material for: Practicability of a Time-Limited Welfare Assessment Protocol for Pasture-Based Dairy Farms, and a Preliminary Assessment of Welfare Outcome Thresholds
Source: Animals (Basel). 2022 Sep 19;12(18):2481. doi: 10.3390/ani12182481 (PMC9495012; doi:10.3390/ani12182481)
Supplement: Supplementary file 1 [file animals-12-02481-s001.zip › animals-1849216-supplementary.pdf]

Supplementry materials

Table S1. Percentage figure for animal-based measures recorded from 23 different pasture-based dairy farms of New Zealand.

Farms	BCS	RFS	Cow cleanliness	Lameness	Skin injury	BT	Coughing
1	4.6	2.0	21.6	1.0	0.3	9.2	1.3
2	3.9	1.9	27.7	1.2	0.2	14.2	1.4
3	4.1	4.3	25.7	2.2	0.0	11.1	1.6
4	8.6	7.8	25.7	0.4	0.0	7.8	1.6
5	7.5	2.2	12.7	0.4	0.4	4.2	1.3
6	4.9	1.8	22.8	0.4	0.4	9.8	2.2
7	2.9	0.1	38.8	6.2	1.0	24.3	0.4
8	1.9	0.0	7.8	2.8	0.0	23.8	0.0
9	14.2	0.0	15.7	2.5	0.2	22.1	1.0
10	2.5	3.1	16.3	2.4	0.6	17.8	0.8
11	3.3	3.3	16.5	1.0	0.2	20.5	0.8
12	1.4	1.7	11.6	1.4	0.6	10.5	1.7
13	3.9	1.6	16.0	4.6	0.4	8.8	0.9
14	2.1	0.0	23.0	3.8	0.0	19.1	1.7
15	1.2	0.6	2.7	2.6	0.2	2.3	1.4
16	2.4	3.1	14.4	3.0	0.4	9.3	1.3
17	3.7	0.0	13.8	1.2	0.2	1.8	1.4
18	4.2	0.0	9.2	2.5	0.0	2.9	0.8
19	5.8	0.0	8.4	9.3	0.4	0.4	1.3
20	4.9	0.1	30.1	5.8	0.1	0.7	0.7
21	11.6	0.8	12.4	3.8	1.3	0.3	1.3
22	6.6	0.0	10.2	2.4	2.4	4.8	0.6
23	5.2	0.9	14.8	6.6	0.7	5.0	0.9

BCS, Body condition scoring below ≤ 3.5 in (%); RFS, Rumen fill score ≤2 in %, BT; Broken tails.

Table S2. Result for measures derived from questionnaire with the farmers from 23 different pasture-based dairy farms.

Farms	Total milking cows	Mortal-ity/yr	Mor-tal-ity%	Farthest paddock (k.m)	Replacement heifer death/yr	Rep %	Lame /yr	Lam e%	Masti-tis/yr	Masti-tis%	Cow mix	Pain re-lief	Vaccina-tion record	Handling aid
1	305	3	1.0	1.2	2	0.7	12	3.9	30	9.8	yes	yes	yes	Plas-tic pipe
2	415	7	1.7	0.8	3	0.7	14	3.4	31	7.5	yes	yes	yes	Plas-tic pipe
3	369	4	1.1	2	3	0.8	15	4.1	25	6.8	yes	yes	yes	no
4	257	0	0.0	1.9	0	0.0	23	8.9	51	19.8	no	yes	yes	no
5	455	2	0.4	1	0	0.0	10	2.2	35	7.7	yes	yes	yes	no
6	224	2	0.9	1	2	0.9	5	2.2	30	13.4	no	yes	yes	no
7	900	25	2.8	2	5	0.6	30	3.3	40	4.4	no	yes	yes	prod, pipe
8	361	4	1.1	1	1	0.3	10	2.8	22	6.1	Yes	yes	yes	pipe, prod
9	485	5	1.0	1.2	3	0.6	15	3.1	60	12.4	No	yes	yes	Rub-ber pipe
10	800	15	1.9	2.6	0	0.0	100	12.5	30	3.8	yes	Yes	yes	plas-tic pipe
11	600	10	1.7	1.5	30	5.0	16	2.7	12	2.0	no	yes	yes	no

12	353	5	1.4	2.5	3	0.8	12	3.4	25	7.1	yes	yes	yes	plastic pipe
13	670	15	2.2	2	20	3.0	26	3.9	90	13.4	no	yes	yes	Plas-tic pipe
14	235	5	2.1	1	1	0.4	10	4.3	7	3.0	no	yes	yes	no
15	658	6	0.9	2.6	2	0.3	36	5.5	20	3.0	no	yes	yes	no
16	700	20	2.9	1.5	2	0.3	120	17.1	150	21.4	yes	yes	yes	no
17	492	10	2.0	2	0	0.0	90	18.3	45	9.1	no	yes	yes	no
18	480	10	2.1	1.5	3	0.6	25	5.2	30	6.3	no	yes	yes	plas-tic pipe
19	225	10	4.4	2	1	0.4	25	11.1	35	15.6	yes	yes	yes	no
20	830	5	0.6	1.8	1	0.1	100	12.0	30	3.6	no	yes	yes	plas-tic pipe
21	372	7	1.9	1	12	3.2	35	9.4	37	9.9	no	yes	yes	no
22	166	1	0.6	1	0	0.0	10	6.0	20	12.0	no	yes	yes	no
23	560	20	3.6	1.5	3	0.5	100	17.9	40	7.1	yes	yes	yes	Rub-ber pipe

File S1. Shade/paddock calculation.

Shade provided by a tree was calculated using height and width. Height of the tree was measured as per (See: How to Estimate the Height of a Tree using a Stick. https://www.youtube.com/watch?v=_kKsp9R9Xb0 (Assessed 1 December 2019)) with pacing used to measure horizontal ground distance . A measuring tape was used to measure the canopy radius of a tree. For farms having trees at the edge/boundaries of the paddocks, only trees providing the shade to the particular paddock were considered in order to calculate shade per paddock. For instance, if a farm had six big trees at the two opposite boundaries, but only three were considered as other three would provide shade to the next paddock.

For the calculation of shade, the total area of the shade provided by the tree in a paddock was calculated with the help of Monteith and Unsworth's (1990) equation as in (Armson, 2012). Here, a is the vertical canopy radius, b is the horizontal canopy radius and β is the angle of the sun to the horizontal. The angle of the sun at the noon was considered to be 70 degrees for uniformity.

$$A_h = \pi b^2 \left\{1 + \frac{a^2}{b^2 \tan^2 \beta}\right\}^{0.5}$$

Here, a is the vertical canopy radius, b is the horizontal canopy radius and β is the angle of the sun to the horizontal. The angle of the sun at the noon was 70 degrees for uniformity.

File S2. Questionnaires related to the records and management-related measures.

a) Distance to the Farthest paddock?
b) Do you mix the cows between the herds? Yes (frequently)…….. No…… Sometimes (upto two times per lactation) ……
c) Do you use pain relief during routinely husbandry procedures? Yes……No….
d) Do you manage your vaccination record? Yes…… No……
e) Do you use aids (for e.g., stick, pipes, prods etc.) for cattle handling? Yes (what?)………… No….
f) What do you use when moving cows from paddock to shed? Dog (Yes/No), Quads (Yes/No),
g) Do you have a single milking herd? Yes…… No….. If 'No' how many herds and what are the herd sizes?...............
h) Number of cows that die on a farm every year......................
i) Number of cows present in a lame cow mob……………….
j) Number of replacement heifers that die before weaning……..
k) Number of lame cows you treat over a year…………………
l) Number of mastitis cases treated per year……………..

Table S3. Results for gate speed and space available per cow in the collecting yard during milking.

Farm	Collecting Yard	Gate speed (m/5s)	Space/cow (m^2/cow)
1	rectangular	0.96	1.47
2	rectangular	1.17	1.77
3	rectangular	1.51	1.32
4	circular	1	1.47
5	circular	1.5	0.89
6	circular	1.37	1.57
7	circular	1	1.08
8	circular	1.4	0.95
9	circular	1.05	1.30
10	circular	1	1.58
11	circular	1	1.98
12	circular	0.46	1.33
13	circular	1	2.01
14	circular	0.83	1.08
15	circular	1.4	1.04
16	circular	1.05	1.29
17	circular	1.25	1.18
18	circular	0.98	2.38
19	circular	1	1.88
20	circular	1.15	1.37
21	circular	0.67	2.09
22	circular	0.5	0.99
23	circular	1	2.12
